# Associations Between Parental Gaming Behaviors and Conversion From Internet Gaming Disorder Noncases to Cases Among Adolescents: Prospective Longitudinal Cohort Study

**DOI:** 10.2196/80061

**Published:** 2026-04-30

**Authors:** Joseph TF Lau, Yanqiu Yu

**Affiliations:** 1 Zhejiang Provincial Clinical Research Center for Mental Disorders, The Affiliated Wenzhou Kangning Hospital, Wenzhou Medical University, Wenzhou, China; 2 Public Mental Health Center, School of Mental Health, Wenzhou Medical University, Wenzhou, China; 3 School of Public Health, Fudan University, 130 Dong'an Road, Shanghai, 200032, China, 86 021-54237707

**Keywords:** internet gaming disorder, parental factors, social cognitive theory, theory of planned behavior, adolescent, longitudinal study

## Abstract

**Background:**

Parental factors are known determinants of internet gaming disorder (IGD) among adolescents. However, the associations between gaming-specific parental factors (eg, parental gaming frequency and parental invitations for cogaming) and IGD have been less investigated, and relevant longitudinal evidence is lacking to inform effective IGD interventions.

**Objective:**

This study aimed to investigate (1) the prevalence of IGD conversion (from a noncase at baseline to a case at follow-up) and (2) the prospective associations between two parental gaming behaviors and IGD conversion as well as their mediation mechanisms via parental supportive attitude toward adolescents’ gaming behaviors and behavioral intention of increasing gaming time.

**Methods:**

A 12-month prospective longitudinal study, with the baseline survey in December 2018 (T1) and the follow-up survey in December 2019 (T2), was conducted among students from 6 convenience-selection junior middle schools in Chengdu and Guangzhou, China. All grade 7 and 8 students of these schools were invited for participation; students self-administered the structured questionnaire on paper and pencil, in classroom settings, and in the absence of schoolteachers. The 9-item DSM-5 Internet Gaming Disorder Symptoms Checklist was used to assess IGD (Cronbach α=0.73). Those with IGD at T1 were excluded, and the final sample size was 2172 (mean age 12.56, SD 0.02, 95% CI 12.52-12.60 years; n=1102, 50.7%, 95% CI 48.7%-52.8% female). The prevalence of IGD conversion was 5.2% (113/2172; 95% CI 4.4%-6.1%).

**Results:**

Adjusted for background factors and respective mediator and outcome scores at T1, two path analysis models showed satisfactory model fit indices (ie, root mean square error approximation=0.02 and 0.03, comparative fit index=0.97 for both models, and standardized root mean square residual=0.02 for both models). The prospective associations between perceived parental gaming frequency and perceived parental invitations for cogaming and IGD conversion were significantly and fully mediated by the 1-mediator indirect path via parental supportive attitude (β=0.02, 95% CI 0.01-0.04 and β=0.02, 95% CI 0.01-0.04) and the 2-mediator indirect path first via parental supportive attitude and then via behavioral intention of increasing gaming time (β=0.006, 95% CI 0.002-0.009 and β=0.005, 95% CI 0.001-0.009) but not the 1-mediator indirect path via the behavioral intention (β=0.01, 95% CI −0.01 to 0.03 and β=0.01, 95% CI −0.01 to 0.03), respectively.

**Conclusions:**

This longitudinal study revealed the prospective associations between 2 parental gaming behaviors and adolescent IGD conversion, as well as the mediation mechanisms, addressing inconsistencies in previous cross-sectional studies and filling in the knowledge gap in longitudinal studies that overlooked parental gaming behaviors. It suggests that parental gaming behaviors may form important family environments shaping adolescents’ perceptions and behaviors related to internet gaming. Family-based prevention and intervention programs on IGD conversion may hence take into account these findings.

## Introduction

Internet gaming disorder (IGD) was classified as “a condition for further study” in Section III of *the Diagnostic and Statistical Manual of Mental Disorders, Fifth Edition* (*DSM-5*) [1] to encourage research into the etiology and treatment of pathological gaming. The clinical relevance was reinforced by the inclusion of gaming disorder in the *International Classification of Diseases, 11th Revision* (*ICD-11*) [2]. Notably, while *ICD-11* focuses on functional impairments, the *DSM-5* operationalizes IGD through 9 cognitive, psychological, and behavioral criteria. This study is grounded in the *DSM-5* framework, focusing on the developmental mechanisms of IGD conversion (ie, the transition from an IGD noncase to a case over time). A meta-analysis reported a pooled global prevalence of IGD of 8.6% among adolescents, with the highest prevalence observed in China (11.7%) [3]. Adolescent IGD was prospectively associated with numerous negative consequences, including depression, anxiety, suicidal ideation, sleep problems, and conduct problems [4-11].

Parental factors are known determinants of adolescent IGD, as parents convey the earliest, strong, and most direct influences on adolescents [12]. This study conceptually categorizes parental factors into 2 domains: general (nonspecific) factors and gaming-specific factors. General factors encompass broad relational constructs, such as parental style, attachment, and parent-child communication [13,14]. In contrast, specific factors refer to parental behaviors directly targeting the child’s gaming activities, such as parental supervision and monitoring [15]. A review highlighted 5 gaming-specific parental behaviors, including no (parental) intervention, cogaming, active mediation (eg, communicating with children without criticism), monitoring (eg, checking children’s gaming without in-depth communication), and restriction (eg, setting rules) [15]. Notably, the literature has focused more on nonspecific parental factors, and there is a dearth of studies investigating modifiable parental gaming behaviors, of which parental gaming frequency and invitations for cogaming (with children) are important. To our knowledge, only 6 cross-sectional studies were located investigating the associations between parental gaming behaviors and adolescent IGD. Three studies reported positive associations among adolescents in mainland [16], Hong Kong [17], and Taiwan [18] China, while the other 3 studies reported nonsignificant results among adolescents in Malaysia [19] and Korea [20,21]. The findings were hence mixed, reflecting the limitations of cross-sectional designs. For instance, the nonsignificant findings might result from the inability to distinguish between parents who game with their children to foster connection (protector) and those whose gaming creates a family environment of excessive gaming (risk). In addition, cross-sectional designs cannot rule out reverse causality (ie, parents might increase cogaming to monitor a child with IGD). Furthermore, although several studies looked at prospective IGD conversion [22-28], none of them involved parental gaming behaviors as predictors. Thus, longitudinal evidence is required to understand the temporal precedence of these parental gaming behaviors in predicting IGD conversion and related mechanisms.

Parental supportive attitude toward the adolescent’s gaming behavior was another parental gaming-specific factor of IGD [15], which may mediate the association between parental gaming behaviors and IGD conversion. According to the theory of cognitive dissonance, parents with gaming behaviors may change their perceptions toward accepting gaming behaviors of themselves and their children to minimize mental discomfort [29]. Hence, parental gaming behaviors may lead to stronger parental supportive attitude toward the adolescent’s gaming behavior (a positive association). In addition, parental supportive attitude might be positively associated with IGD conversion, as it forms a subjective norm supporting adolescent gaming. Subjective norm is a key construct of the theory of planned behavior (TPB) [30], postulating that behavioral attitude, subjective norm, and perceived behavioral control would determine behavioral intention, which would in turn determine the actual behavior (gaming behavior in this case). Parental supportive attitude may hence mediate the associations between parental gaming behaviors and IGD conversion.

Plausibly, behavioral intention of increasing gaming time would further mediate between parental supportive attitude and IGD conversion, as TPB implies that parental supportive attitude would increase adolescents’ behavioral intention of increasing gaming time, which may in turn increase IGD. Behavioral intention of increasing gaming time was a significant predictor of adolescent IGD [30], and the reduction in gaming time has been commonly used as an intervention component and/or goal in IGD interventions [31,32]. However, to our knowledge, no study has investigated whether behavioral intention of increasing gaming time predicts IGD conversion. Parental gaming behaviors may also increase adolescents’ behavioral intention of increasing gaming time, heightening the risk of IGD conversion. Thus, it is contended that behavioral intention of increasing gaming time would mediate the associations between unfavorable parental gaming behaviors and parental supportive attitude and IGD conversion.

The associations between parental gaming behaviors and parental supportive attitude and IGD conversion were further supported by the widely used social cognitive theory (SCT) [12]. First, the observational learning construct postulates that an individual performs a behavior by observing and imitating his or her role models [12]. Accordingly, adolescents may learn to game more by observing their parents’ gaming behaviors, increasing the risk of IGD conversion. Second, according to the reciprocal determinism construct, health behaviors are determined by the interactions among environmental, personal, and behavioral factors [12]. Doubtlessly, parental supportive attitude is a salient feature of the family environment [12], which would affect IGD conversion.

Given the background, this prospective longitudinal study aimed to (1) investigate the prevalence of IGD conversion (ie, from non-IGD cases at baseline [T1] to IGD cases at follow-up [T2]) over a 12-month follow-up period among adolescents in China and (2) examine the mediation effects of parental supportive attitude toward the adolescent’s gaming behavior and adolescents’ behavioral intention of increasing gaming time on the associations between parental gaming behaviors (ie, perceived parental gaming frequency and perceived parental invitation for cogaming) and IGD conversion. It was hypothesized that (1) perceived parental gaming frequency (T1) would be prospectively and positively associated with parental supportive attitude (T2) that would be positively associated with IGD conversion (T2), (2) perceived parental gaming frequency (T1) would be prospectively and positively associated with adolescents’ behavioral intention of increasing gaming time (T2) that would be positively associated with IGD conversion (T2), and (3) perceived parental gaming frequency (T1) would be prospectively and positively associated with parental supportive attitude (T2) that would be positively associated with adolescents’ behavioral intention of increasing gaming time (T2), which would be positively associated with IGD conversion (T2). Similar research hypotheses were generated with the variable of perceived parental gaming frequency replaced by perceived parental invitation for cogaming.

## Methods

### Participants and Procedures

A 12-month prospective longitudinal cohort study was conducted in Guangzhou and Chengdu, China, which had population sizes of 18.3 and 16.6 million, respectively. The baseline survey and follow-up survey were conducted in December 2018 (T1) and December 2019 (T2), respectively. Four junior middle schools from Guangzhou and 2 from Chengdu were selected by convenience sampling. All grade 7 and 8 (7 and 8 years of formal schooling) students of those schools were invited to join this study. Grade 9 students were not invited as they would leave the school before the end of the 12-month follow-up period. As we were investigating IGD conversion, those with IGD at baseline were removed from data analysis (an exclusion criterion). The structured questionnaire was self-administered anonymously using paper and pencil, in the classroom setting, and in the absence of the schoolteachers. Well-trained fieldworkers briefed the students about the objective, content, logistics, and voluntary nature of the study. The reporting of this study follows the Journal Article Reporting Standards for Studies Using Structural Equation Modeling [33].

### Ethical Considerations

The ethics approval of this project was obtained from the Survey and Behavioral Research Ethics Committee of the Chinese University of Hong Kong (SBRE-18‐430). This study used a parental opt-out procedure, and student participation was strictly voluntary and uncompensated. Due to the anonymous study design, written informed consent was waived; instead, the act of completing and returning the questionnaire indicated informed consent, which was explicitly stated on the cover page of the questionnaire. To facilitate longitudinal data matching while guaranteeing anonymity, a unique matching code was used combining participants’ birthdays, the last 4 digits of their parents’ mobile phone numbers, and the last 2 letters of their parents’ given names. Consequently, no personally identifiable information was collected, and no identifiable information could be identified in both the datasets analyzed and this manuscript. No incentives were provided to participants, parents, and schoolteachers in this study.

### Measurements

#### Background Factors Assessed at T1

The information included sex, age, city, whether participants had moved to the city, parental educational levels (junior middle school or below, senior middle school, or college or above), and perceived family financial situation (very good, good, moderate, poor, or very poor). These variables, except for age, were treated as categorical variables in data analysis.

#### Perceived Parental Gaming Behaviors Assessed at T1

Perceived parental gaming frequency was assessed by the item, *“*How often do your parents play internet games (1=never to 4=always; a continuous variable)?” Parental invitation for cogaming was assessed by the item, “How often do your parents invite you to play internet games together (1=never to 4=always; a continuous variable)?” Both items have been applied to Chinese adolescents in extant literature [16-18].

#### Adolescent IGD Conversion at T2

The 9-item *DSM-5* checklist was used to assess IGD [1]. It recorded the presence of 9 addictive symptoms, including preoccupation, withdrawal, tolerance, inability to control gaming, loss of interest in other activities, psychological and/or social problems, deception, avoidance, and significant loss due to gaming. IGD was defined by the endorsement of 5 or more items in the past 12 months (yes or no responses) [34]. The Chinese version of the *DSM-5* has been validated in adolescents with satisfactory psychometric properties [35]. The Cronbach α of the checklist was 0.73 at T2 in this study. This variable assessed IGD conversion from T1 to T2 and was treated as a binary variable.

#### Perceived Parental Supportive Attitude Toward the Adolescent’s Gaming Behavior at T2

Parental supportive attitude toward the adolescent’s gaming behavior was assessed using the item, “To what extent do your parents support your gaming behavior (1=strongly unsupportive to 5=strongly supportive; a continuous variable)?”

#### Behavioral Intention of Increasing Gaming Time at T2

Behavioral intention of increasing gaming time was assessed by the item, “Do you intend to increase or reduce your gaming time in the next year (5=intend to increase substantially; 4=intend to increase slightly/moderately; 3=no intention to change; 2=intend to reduce slightly/moderately; 1=intention to reduce substantially; a continuous variable)?”

### Sample Size Planning

As path analysis was used as the key statistical method, the rule of thumb for its sample size planning requires a minimum of 10 cases per item or an observed variable [36]. Accordingly, this study requires a sample size of at least 140 participants, and the current sample size is adequate.

### Statistical Analysis

Attrition analysis was conducted by examining between-group differences in the background variables and 2 parental gaming behaviors at T1 between follow-ups and dropouts. The Little missing completely at random (MCAR) test was conducted to evaluate the pattern of missing data due to dropouts; multiple imputation (n=20) was used to address missing data. Descriptive statistics and normality tests (via the Kolmogorov-Smirnov and Shapiro-Wilk tests) were performed for the key studied variables. Spearman correlation coefficients were derived for the correlations between two parental gaming behaviors (T1), parental supportive attitude (T2), behavioral intention of increasing gaming time (T2), and IGD conversion (T2). Path analysis was performed to test the hypothesized mediation mechanisms, with the weighted least square mean and variance adjusted estimator. The models were adjusted for all studied background factors and respective baseline levels of mediators and outcomes (ie, parental supportive attitude at T1 for parental supportive attitude at T2). Satisfactory model fit indices for the path analysis models included comparative fit index ≥0.90, root mean square error of approximation ≤0.08, and standardized root mean square residual ≤0.08. Path analysis was performed by using Mplus (version 7.0; Muthén & Muthén) and other analyses by SPSS (version 21.0; IBM Corp). Statistical significance was indicated as *P*<.05 and 95% CI not involving zero.

## Results

### Participant Characteristics

**Figure 1.**
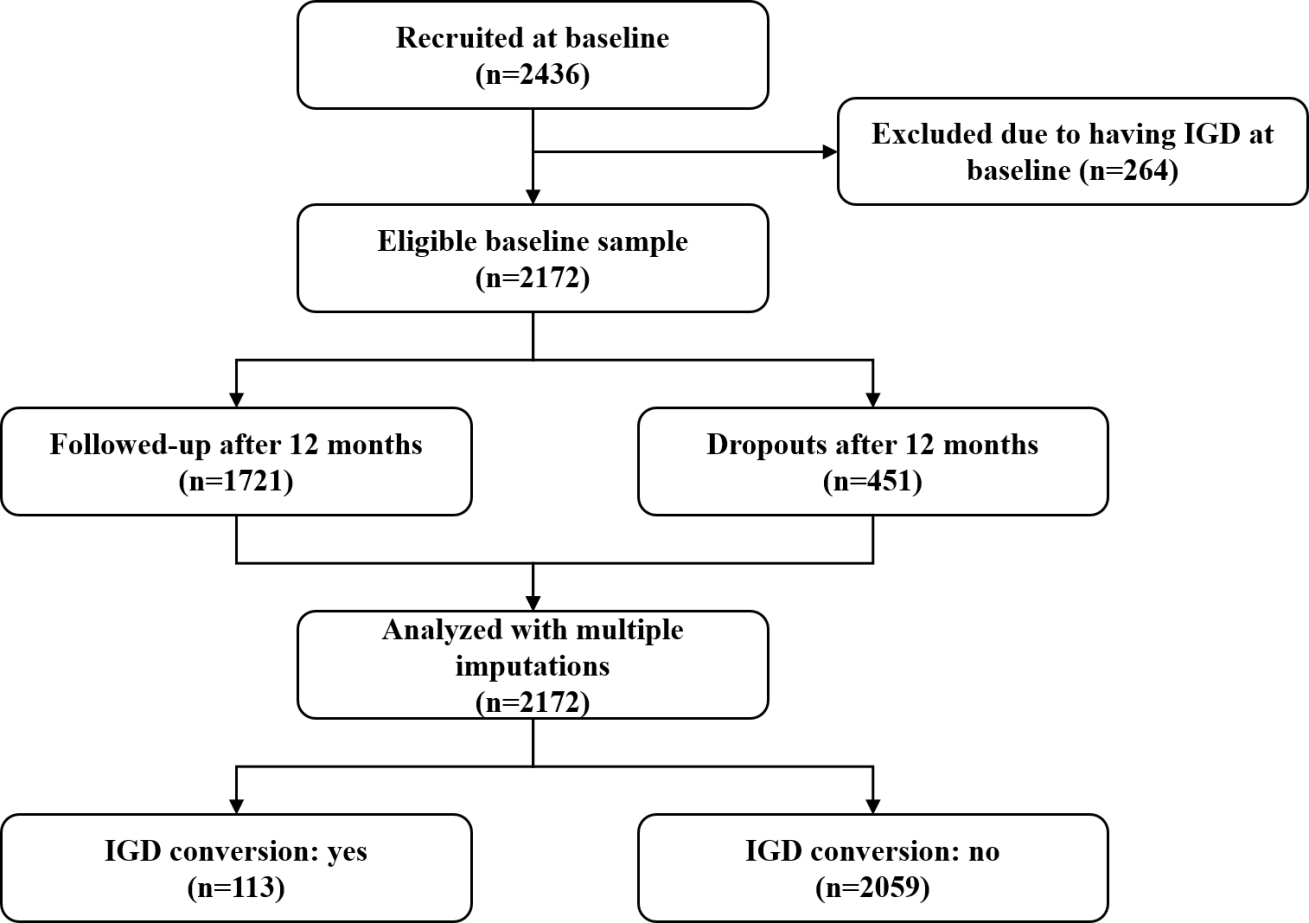
Flowchart of the 2-wave longitudinal study on mediation mechanism between parental gaming behaviors and internet gaming disorder (IGD) conversion among adolescents from 2 Chinese cities from December 2018 to December 2019.

A total of 2436 students completed the baseline questionnaire; 264 (10.8%) with IGD at T1 were removed. Of the remaining 2172 participants, 1721 (79.2%) were matched for their baseline and follow-up questionnaires (Figure 1). Those lost to follow-up were more likely to be a Chengdu student, older, female, not having moved to the city, and having parental educational levels of junior middle school or below; their parents also played internet games and invited them to cogame more frequently (Table 1). The Little MCAR test was statistically significant (*χ*^2^_302_=599.5; *P*<.001), suggesting that data were not missing completely at random.

**Table 1.** Attrition analysis of the 2-wave longitudinal study on mediation mechanism between parental gaming behaviors and internet gaming disorder (IGD) conversion among 2172 adolescents from 2 Chinese cities from December 2018 to December 2019.

	Followed up (n=1721)		Lost to follow-up (n=451)		*P* value^a^
Study city, n (%; 95% CI)					<.001
Guangzhou	1100 (63.9; 61.6-66.2)		150 (33.3; 28.9-37.8)		
Chengdu	621 (36.1; 33.8-38.4)		301 (66.7; 62.2-71.1)		
Age (y)^b^, mean (SD; 95% CI)	12.54 (0.7; 12.51-12.58)		12.8 (0.7; 12.7-12.9)		<.001
Sex, n (%; 95% CI)					.002
Female	800 (46.5; 44.1-48.9)		251 (55.7; 51.1-60.1)		
Male	888 (51.6; 49.2-54.0)		195 (43.2; 38.7-47.9)		
Unknown	33 (1.9; 1.4-2.7)		5 (1.1; 0.4-2.7)		
Whether moving to the city, n (%; 95% CI)					<.001
No	288 (17.1; 15.0-18.6)		108 (24.8; 20.1-28.2)		
Yes	1393 (82.9; 79.0-84.6)		328 (75.2; 68.4-76.8)		
Father’s educational level, n (%; 95% CI)					<.001
Junior middle school or below	664 (38.6; 36.3-40.9)		229 (50.8; 46.1-55.5)		
Senior middle school	454 (26.4; 24.3-28.5)		129 (28.6; 24.5-33.0)		
College or above	489 (28.4; 26.3-30.6)		58 (12.9; 9.9-16.3)		
Unknown	114 (6.6; 5.5-7.9)		35 (7.8; 5.5-10.6)		
Mother’s educational level, n (%; 95% CI)					<.001
Junior middle school or below	678 (39.4; 37.1-41.7)		233 (51.7; 46.9-56.4)		
Senior middle school	475 (27.6; 25.5-29.8)		116 (25.7; 21.7-30.0)		
College or above	456 (26.5; 24.4-28.6)		63 (14; 10.9-17.5)		
Unknown	112 (6.5; 5.4-7.8)		39 (8.6; 6.2-11.6)		
Perceived family financial situation, n (%; 95% CI)					.29
Good or very good	463 (26.9; 24.8-29.1)		109 (24.2; 20.3-28.4)		
Moderate	1055 (61.3; 59.0-63.6)		286 (63.4; 58.8-67.9)		
Poor or very poor	168 (9.8; 8.4-11.3)		51 (11.3; 8.5-14.6)		
Unknown	35 (2; 1.4-2.8)		5 (1.1; 0.4-2.6)		
Perceived parental gaming frequency (range 1 to 4), mean (SD; 95% CI)	1.63 (0.8; 1.60-1.67)		1.8 (0.9; 1.7-2.0)		.003
Perceived parental invitation for cogaming (range 1 to 4), mean (SD; 95% CI)	1.18 (0.5; 1.15-1.20)		1.4 (0.6; 1.3-1.5)		<.001

a Chi-square tests and independent-sample *t* tests were used as applicable.

b Mean and SD are reported for continuous variables. Percentages are calculated within each follow-up group (followed up and lost to follow-up).

Of the 2172 participants based on the 20 imputed dataset, the mean age was 12.56 (SD 0.02, 95% CI 12.52-12.60) years. More than half were female (n=1102, 50.7%, 95% CI 48.7%-52.8%); about two-fifths of their fathers (n=953, 43.9%, 95% CI 41.8%-46.0%) and mothers (n=976, 44.9%, 95% CI 42.8%-47.0%) had received junior middle school or below education. Close to one-tenth self-reported a poor or very poor family financial situation (n=223, 10.3%, 95% CI 9.0%-11.7%). The prevalence of IGD conversion over a 12-month follow-up period was 5.2% (n=113, 95% CI 4.4%-6.1%; Table 2). The mean scores were 1.66 (SD 0.02; 95% CI 1.63-1.69; range 1‐4) for perceived parental gaming frequency, 1.21 (SD 0.01, 95% CI 1.19-1.23; range 1‐4) for perceived parental invitation for cogaming, 2.01 (SD 0.02, 95% CI: 1.96-2.06; range 1‐5) for perceived parental supportive attitude, and 1.91 (SD 0.02, 95% CI 1.87-1.95; range 1‐5) for behavioral intention of increasing gaming time.

**Table 2.** Participant characteristics of the 2-wave longitudinal study on mediation mechanism between parental gaming behaviors and internet gaming disorders (IGD) conversion among 2172 adolescents from 2 Chinese cities from December 2018 to December 2019. The frequencies and percentages represent pooled estimates after multiple imputation.

Background factors	Participants, n (%; 95% CI)	
Study city		
Guangzhou	1250 (57.6; 55.5-59.6)	
Chengdu	922 (42.4; 40.4-44.5)	
Sex		
Male	1070 (49.3; 47.2-51.3)	
Female	1102 (50.7; 48.7-52.8)	
Whether moving to live in the studied city		
No	406 (18.7; 17.2-20.3)	
Yes	1766 (81.3; 79.7-82.8)	
Father’s educational level		
Junior middle school or below	953 (43.9; 41.8-46.0)	
Senior high school or equal	627 (28.9; 27.0-30.9)	
College or above	592 (27.3; 25.4-29.2)	
Mother’s educational level		
Junior middle school or below	976 (44.9; 42.8-47.0)	
Senior high school or equal	637 (29.3; 27.4-31.3)	
College or above	559 (25.7; 23.9-27.7)	
Perceived family financial situation		
Good or very good	585 (27; 25.1-28.9)	
Moderate	1364 (62.8; 60.8-64.8)	
Poor or very poor	223 (10.3; 9.0-11.7)	
IGD conversion (T2^a^)		
No	2059 (94.8; 93.9-95.6)	
Yes	113 (5.2; 4.4-6.1)	

a T2: assessed at follow-up.

### Correlation Analysis

Kolmogorov-Smirnov and Shapiro-Wilk tests showed that all studied key variables violated the normality assumption (all *P*<.001). Spearman correlation analysis showed that perceived parental gaming frequency, perceived parental invitation for cogaming, parental supportive attitude, and behavioral intention of increasing gaming time were significantly and positively correlated with IGD conversion. Perceived parental gaming frequency and parental supportive attitude were positively correlated with behavioral intention of increasing gaming time, but the correlation between parental invitation for cogaming and behavioral intention was statistically nonsignificant. The 3 variables related to parental gaming behavior and attitudes were positively correlated with each other (Table 3).

**Table 3.** Spearman correlations among the key variables of the 2-wave longitudinal study on mediation mechanisms between parental gaming behaviors and internet gaming disorder (IGD) conversion among 2172 adolescents from 2 Chinese cities from December 2018 to December 2019.

	Parental gaming frequency (T1^a^), ρ (95% CI)	Parental invitation for cogaming with children (T1), ρ (95% CI)	Parental supportive attitude toward adolescents’ gaming behavior (T2^b^), ρ (95% CI)	Behavioral intention of increasing gaming time (T2), ρ (95% CI)
Parental gaming frequency (T1)	1.00	—^c^	—	—
Parental invitation for cogaming with children (T1)	0.37 (0.34-0.41)	1.00	—	—
Parental supportive attitudes toward the adolescent’s gaming behavior (T2)	0.13 (0.08-0.17)	0.07 (0.03-0.11)	1.00	—
Behavioral intention of increasing gaming time (T2)	0.07 (0.03-0.12)	0.04 (0.002-0.09)	0.13 (0.09-0.17)	1.00
IGD conversion (T2)	0.05 (0.01-0.09)	0.05 (0.01-0.09)	0.05 (0.01-0.10)	0.11 (0.07-0.15)

a T1: assessed at baseline.

b T2: assessed at follow-up.

c Not applicable.

### Path Analysis

**Figure 2.**
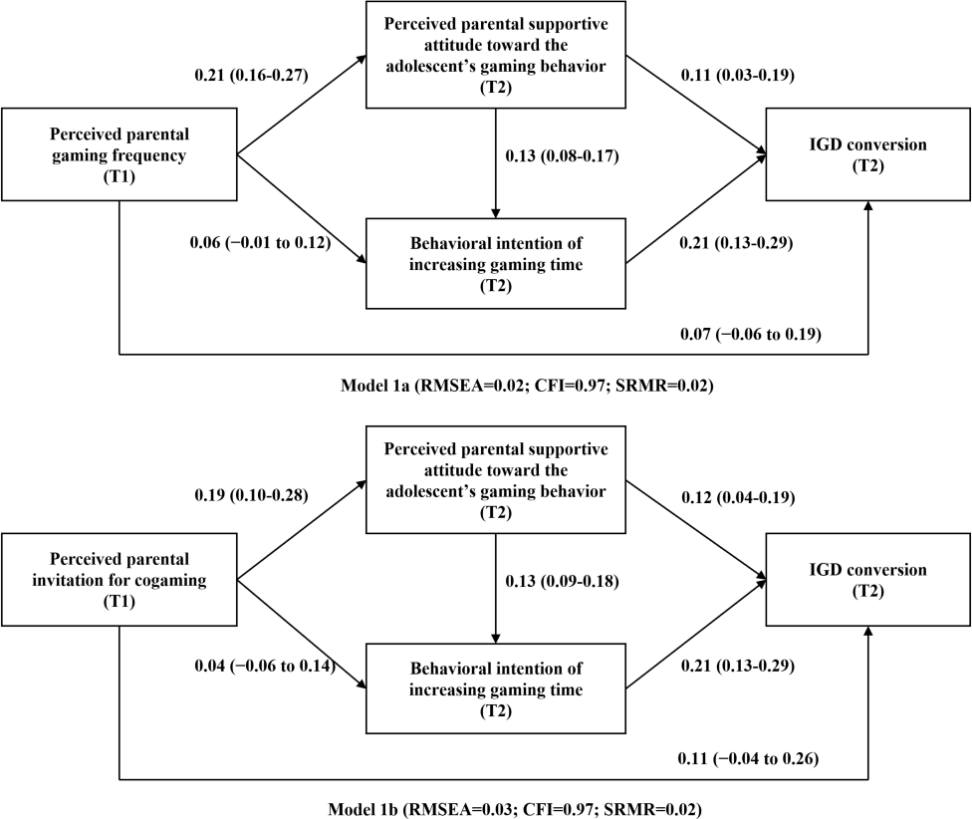
Path analysis models testing the mediation mechanism between parental gaming behaviors and internet gaming disorder (IGD) conversion in the 2-wave longitudinal study among 2172 adolescents from 2 Chinese cities from December 2018 to December 2019. Standardized coefficients were reported as β (95% CI). The models were adjusted for city, age, sex, whether participants had moved to the city, fathers’ and mothers’ educational level, perceived family financial situation, and respective baseline levels of the mediators and the outcome. CFI: comparative fit index; RMSEA: root mean square error of approximation; SRMR: standardized root mean square residual; T1: assessed at baseline; T2: assessed at follow-up.

Figure 2A presents path analysis testing the mediation effects of parental supportive attitude and behavioral intention of increasing gaming time on the association between perceived parental gaming frequency and IGD conversion. The 1-mediator indirect path via parental supportive attitude *(*β=0.02, 95% CI 0.01-0.04) and the 2-mediator indirect path (first via parental supportive attitude then via behavioral intention; β=0.006, 95% CI 0.002-0.009) were statistically significant, whereas the 1-mediator indirect path via behavioral intention (β=0.01, 95% CI −0.01 to 0.03) was not. As the association between perceived parental gaming frequency and IGD conversion was statistically nonsignificant (β=0.07, 95% CI −0.06 to 0.19), full mediation via the 2 indirect paths was observed. Similar patterns were observed for the mediation between perceived parental invitation for cogaming and IGD conversion (Figure 2). The 1-mediator indirect path via parental supportive attitude (β=0.02, 95% CI 0.01-0.04) and the 2-mediator indirect path (first via parental supportive attitude then via behavioral intention; β=0.005, 95% CI 0.001-0.009) were statistically significant, whereas the 1-mediator indirect path via behavioral intention of increasing gaming time (β=0.01, 95% CI −0.01 to 0.03) was not. As the association between perceived parental invitation for cogaming and IGD conversion was also statistically nonsignificant (β=0.11, 95% CI −0.04 to 0.26), full mediation via the 2 indirect paths was observed. Both models support the original hypothesized models, and the other relevant statistics are presented in Multimedia Appendix 1.

## Discussion

### Principal Findings

This longitudinal study revealed the prospective associations between 2 parental gaming-specific factors of perceived parental gaming frequency and parental invitations for cogaming at T1 and IGD conversion at T2 among Chinese adolescents. Furthermore, the 2 individual path analysis models revealed that these prospective associations were fully mediated by a 2-mediator indirect path (first via parental supportive attitude toward the adolescent’s gaming behavior and then via adolescents’ behavioral intention of increasing gaming time) and the 1-mediator indirect path via parental supportive attitude, but not the other 1-mediator indirect path via behavioral intention. The results have implications for IGD interventions involving parents.

This study documented the prevalence of IGD conversion of 5.2% (113/2172) among previously non-IGD adolescents in China, which was slightly lower than 11.7% [22] and 1.7% [37] among participants aged 14 years or older in Western countries. Our results identify 2 parental gaming behaviors positively associated with IGD conversion among Chinese adolescents, highlighting the role of environmental triggers [12]. Specifically, higher frequencies of parental gaming and invitations for cogaming may serve as environmental endorsements of adolescent gaming and facilitate their IGD conversion. Aside from the observational learning perspective of SCT, these parental gaming behaviors can be cues to action, prompting intensive gaming that may increase adolescent IGD conversion [38]. Hence, health education should target not only adolescents but also their parents. Behavioral intention of increasing gaming time was also positively associated with IGD conversion, supporting TPB that behavioral intentions predict corresponding health behaviors [30]. A behavioral intention is often not translated into the actual behavior [39], but this “intention-behavior gap” could be reduced by implementation planning interventions [40]. For instance, the if-then intervention (“if” facing temptation and “then” implementing a mentally rehearsed preventive response) [40] has demonstrated strong effectiveness in achieving behavioral change goals [41].

In addition, the significant indirect paths shed insights into how to reduce IGD conversion. Both parental gaming behaviors were prospectively associated with parental supportive attitude that was positively associated with behavioral intention of increasing gaming time, which was associated with a higher risk of IGD conversion. The observed full mediation effects suggest that the mediation mechanism via parental supportive attitude alone and first via parental supportive attitude and then via behavioral intention of increasing gaming time may fully explain the associations between parental gaming behaviors at T1 and IGD conversion at T2. Notably, although both indirect paths reached statistical significance, the magnitudes of the involved associations were small. It is important to acknowledge that the statistical significance of these paths may be due to the large sample size of this study [42]. These small effect sizes reflect the multidimensional etiology of IGD conversion [43], wherein parental gaming behaviors and attitudes and personal cognition (behavioral intention) account for only a minute portion of the overall variance. Accordingly, while these variables (eg, parental gaming behaviors) are widespread and modifiable [12], they are better conceptualized as small, interacting components within a broader biopsychosocial network of risk factors, rather than stand-alone targets. This suggests that public health interventions should not rely on targeting single environmental factors but instead adopt holistic, multicomponent approaches that integrate family-level determinants alongside other individual and systemic factors. An additional remark is that the 1-mediator indirect path from parental gaming behaviors to IGD conversion via behavioral intention alone was statistically nonsignificant. This suggests that parental gaming behaviors were prospectively and directly associated with parental supportive attitude, but not behavioral intention, and that the association between parental supportive attitude and IGD conversion was mediated via behavioral intention of increasing gaming time. As TPB postulates that the association between attitude and behavior would be mediated via behavioral intention [30], this finding supports the TPB.

Overall, the earlier findings offer practical implications that prevention of IGD conversion should not neglect both parental and adolescent gaming behaviors and attitudes. Inevitably, parental gaming behaviors (eg, cogaming with children) and parental attitude are a double-edged sword, as they may both increase the risk of adolescent IGD [16-18] and improve parent-child relationships [44]. A delicate balance needs to be reached. Parents may use their gaming behaviors to set good examples in time management, nonexcessive gaming, and self-control for their children. Although excessive cogaming may be problematic, cogaming is a good opportunity for parent-child communication on healthy gaming [15]. Parents should be informed that parental supervision and monitoring are protective factors against adolescent IGD [14] and that parental supportive attitude toward the adolescent’s gaming behavior needs to be carefully conveyed to the children to avoid the wrong impression of absent parental supervision and monitoring. Hence, effective family-based IGD interventions on health education, parenting skills, and improving family relationships [45,46] may consider integrating these findings.

This study has several limitations. First, the attrition rate was 22.6%, and there were significant differences between the retained participants and dropouts. Specifically, those dropouts reported higher frequencies of parental gaming and invitations for cogaming. Furthermore, the MCAR test confirmed that the data were not missing completely at random. To reduce this selection bias and improve the validity of the estimates, multiple imputation was used in data analysis to account for these missing data patterns [47]. Second, convenience sampling may introduce sampling bias [48]. As there might be geographic variations, the generalizability of these findings to other regions and countries should be made with caution. Third, as the questionnaire was self-administered, social desirability bias might be present [48], and several measures were taken to mitigate this risk. The survey was anonymous, and no schoolteachers were present in the classroom during administration; data collection was managed entirely by external research staff to encourage honest reporting. Another notable limitation is the single-item measures to assess parental supportive attitude and behavioral intention of increasing gaming time. While single-item measures can reduce participant burden and are considered valid for concrete constructs [49], they preclude the estimation of internal consistency reliability. As path analysis assumes that observed variables are measured without error [50], the presence of potential random measurement errors in these single items may underestimate path coefficients [51]. Future studies should verify these findings using validated multi-item scales. Furthermore, IGD was assessed by the 9-item *DSM-5* IGD checklist instead of the *ICD-11* gaming disorder criteria, as the former was the prevailing diagnostic standard for large-scale epidemiological studies at the time of baseline data collection (December 2018). Although the clinical validity of the *DSM-5* IGD checklist has been validated in Chinese populations [34], this self-report screening tool may overestimate IGD prevalence compared with a clinical gold standard administered by a psychiatrist [52]. Furthermore, this prospective study used a 2-wave design, and both mediators and IGD conversion were assessed concurrently at T2. Although the baseline variables were adjusted, this design precludes the establishment of a complete temporal relationship (ie, predictor to mediator to outcome). As such, the specific causal relationships between the mediators and IGD conversion cannot be statistically confirmed and should be interpreted as associations within a prospective framework rather than causal mechanisms. Finally, this study did not include data on gaming time or frequency, which are known predictors of IGD conversion [43]; this limits the ability to assess whether the observed associations exist independently of gaming time. Furthermore, this study did not assess peer factors, which are important determinants of adolescent gaming [17]. This limits the ability to compare the relative strength of parental versus peer influences. Future research should integrate peer variables to provide a more comprehensive ecological model of IGD conversion.

### Conclusions

In conclusion, this longitudinal study is novel to identify the prospective associations between specific parental gaming behaviors and the risk of IGD conversion among Chinese adolescents. Grounded in SCT and TPB, these findings elucidate the mediation mechanisms involving family environment and individual cognitive processes. Specifically, the identification of parental supportive attitude toward the adolescent’s gaming behavior and adolescents’ behavioral intention of increasing gaming time as key mediators reveals a nuanced parental behavior–adolescent cognition pathway, suggesting that parental modeling not only provides a behavioral template but also shapes the internal cognitive appraisals and behavioral intentions that precede IGD conversion.

These results bear theoretical and practical implications. Theoretically, they extend the application of SCT and TPB to the field of behavioral addiction by highlighting how distal environmental factors (parental behavior) are internalized through proximal cognitive factors. Practically, the findings underscore the necessity of shifting the focus of IGD prevention from solely adolescent-centered approaches to family-based interventions. Prevention strategies should involve educating parents on their own gaming habits and fostering healthier cognitive appraisals of gaming within the household. Moreover, mitigating the risk of adolescent IGD conversion requires a holistic approach that targets both modifiable parental influences and the subsequent cognitive distortions in adolescents.

## Supplementary material

10.2196/80061 Multimedia Appendix 1 Path analysis testing the mediation mechanism between parental gaming behaviors and IGD conversion in the 2-wave longitudinal study among 2172 adolescents from 2 Chinese cities from December 2018 to December 2019.

## Abbreviations:

*DSM-5*: *Diagnostic and Statistical Manual of Mental Disorders* (*Fifth Edition*)
*ICD-11*: *International Classification of Diseases, 11th Revision*
IGD: internet gaming disorder
MCAR: missing completely at random
SCT: social cognitive theory
TPB: theory of planned behavior
