# Supplementary material for: Associations Between Parental Gaming Behaviors and Conversion From Internet Gaming Disorder Noncases to Cases Among Adolescents: Prospective Longitudinal Cohort Study
Source: JMIR Serious Games. 2026 Apr 30;14:e80061. doi: 10.2196/80061 (PMC13131833; doi:10.2196/80061)
Supplement: Multimedia Appendix 1 [file games-v14-e80061-s001.docx]

Table S1. Path analysis testing the mediation mechanism between parental gaming behaviors and IGD conversion in the 2-wave longitudinal study among 1594 adolescents from two Chinese cities from December 2018 to December 2019

|  | Unstandardized coefficients  (95% CI) | S.E. | Standardized coefficients  (95% CI) | S.E. |
| --- | --- | --- | --- | --- |
| **Model a** (*df*=6; RMSEA=0.02; CFI=0.97; SRMR=0.02) |  |  |  |  |
| ***Structural paths*** |  |  |  |  |
| Perceived parental gaming frequency → PSA-AGB | 0.21 (0.15, 0.27) | 0.03 | 0.21 (0.16, 0.27) | 0.03 |
| Perceived parental gaming frequency → BI-IGT | 0.05 (−0.01, 0.11) | 0.03 | 0.06 (−0.01, 0.12) | 0.03 |
| PSA-AGB → BI-IGT | 0.11 (0.07, 0.15) | 0.02 | 0.13 (0.08, 0.17) | 0.02 |
| Perceived parental gaming frequency → IGD conversion | 0.07 (−0.06, 0.20) | 0.07 | 0.07 (−0.06, 0.19) | 0.06 |
| PSA-AGB → IGD conversion | 0.12 (0.04, 0.20) | 0.04 | 0.11 (0.03, 0.19) | 0.04 |
| BI-IGT → IGD conversion | 0.25 (0.15, 0.35) | 0.05 | 0.21 (0.13, 0.29) | 0.04 |
| *Indirect paths* |  |  |  |  |
| Perceived parental gaming frequency → PSA-AGB → IGD conversion | 0.03 (0.01, 0.04) | 0.01 | 0.02 (0.01, 0.04) | 0.01 |
| Perceived parental gaming frequency → BI-IGT → IGD conversion | 0.01 (−0.01, 0.03) | 0.01 | 0.01 (−0.01, 0.03) | 0.01 |
| Perceived parental gaming frequency → PSA-AGB → BI-IGT → IGD conversion | 0.006 (0.002, 0.009) | 0.002 | 0.006 (0.002, 0.009) | 0.002 |
|  |  |  |  |  |
| **Model b** (*df*=6; RMSEA=0.03; CFI=0.97; SRMR=0.02) |  |  |  |  |
| ***Structural paths*** |  |  |  |  |
| Perceived parental invitation for co-gaming → PSA-AGB | 0.19 (0.09, 0.27) | 0.05 | 0.19 (0.10, 0.28) | 0.05 |
| Perceived parental invitation for co-gaming → BI-IGT | 0.03 (−0.06, 0.12) | 0.05 | 0.04 (−0.06, 0.14) | 0.05 |
| PSA-AGB → BI-IGT | 0.12 (0.08, 0.16) | 0.02 | 0.13 (0.09, 0.18) | 0.02 |
| Perceived parental invitation for co-gaming → IGD conversion | 0.12 (−0.04, 0.27) | 0.08 | 0.11 (−0.04, 0.26) | 0.08 |
| PSA-AGB → IGD conversion | 0.12 (0.04, 0.20) | 0.04 | 0.12 (0.04, 0.19) | 0.04 |
| BI-IGT → IGD conversion | 0.25 (0.15, 0.35) | 0.05 | 0.21 (0.13, 0.29) | 0.04 |
| *Indirect paths* |  |  |  |  |
| Perceived parental invitation for co-gaming → PSA-AGB → IGD conversion | 0.02 (0.01, 0.04) | 0.01 | 0.02 (0.01, 0.04) | 0.01 |
| Perceived parental invitation for co-gaming → BI-IGT → IGD conversion | 0.01 (−0.01, 0.03) | 0.01 | 0.01 (−0.01, 0.03) | 0.01 |
| Perceived parental invitation for co-gaming → PSA-AGB → BI-IGT → IGD conversion | 0.005 (0.002, 0.009) | 0.002 | 0.005 (0.001, 0.009) | 0.002 |

Note. PSA-AGB=Perceived parental supportive attitude toward the adolescent’s gaming behavior; BI-IGT=Behavioral intention of increasing gaming time; IGD=Internet gaming disorder. The models were adjusted for city, age, sex, whether moving to the city, fathers’ and mothers’ educational level, perceived family financial situation, and respective baseline levels of the mediators and the outcome.
